# Stress and anxiety in parents of mentally retarded children

**DOI:** 10.4103/0019-5545.55937

**Published:** 2005

**Authors:** Mita Majumdar, Yvonne Da Silva Pereira, John Fernandes

**Affiliations:** * Psychologist, Institute of Psychiatry and Human Behaviour, Bambolim, Goa 403202; ** Lecturer, Institute of Psychiatry and Human Behaviour, Bambolim, Goa 403202; *** Professor, Institute of Psychiatry and Human Behaviour, Bambolim, Goa 403202

**Keywords:** Mental retardation, anxiety, stressors

## Abstract

**Background:**

Studies comparing the stress perceived by parents of mentally retarded and normal children are limited.

**Aim:**

(i) To find whether there exists a difference in the perceived stress between both the parents of mentally retarded children, (ii) to study whether these stresses occur more frequently in parents of mentally retarded children compared with those of normal children, and (iii) to find any correlation between the severity of perceived stressors and the anxiety state of these parents.

**Methods:**

This study was conducted in the Child Guidance Clinic of a tertiary care psychiatry hospital. The study sample, comprising 180 subjects, was categorized as: group A (60 parents of profound to moderately mentally retarded children), group B (60 parents of mild to borderline mentally retarded children) and group C (60 parents of children with normal intelligence), which served as the control group. Each parent was evaluated using the Family Interview for Stress and Coping (FISC) in Mental Retardation, and the Hamilton Anxiety Rating Scale (HARS).

**Results:**

Parents in group A had a significantly higher frequency of stressors and level of anxiety as compared to those in groups B and C. A positive correlation was found between the level of anxiety and stressors.

**Conclusion:**

Multifaceted factors made parents in groups A and B more vulnerable to stress compared with parents in the control group.

## INTRODUCTION

A mentally retarded child in a family is usually a serious stress factor for the parents. It often requires a reorientation and reevaluation of family goals, responsibilities and relationships. In India, the majority of persons with mental retardation have traditionally been cared for by their families. In today's modern society this home-based care has resulted in many adverse consequences. Factors such as changes in the social system (e.g. breaking up of joint families) and the economic system (e.g. unemployment, inflation, etc.) have contributed to the stress that parents of mentally retarded children experience.

The emotional and social stress that these parents undergo have been described by various investigators in the East and West.^1^–^5^ On the other hand, few studies have shown that stress is not an inevitable consequence in these parents.^6^,^7^ However, studies comparing the stress perceived by parents of mentally retarded and normal children are limited. Therefore, we undertook this study (i) to find any difference in the perceived stress between both the parents of mentally retarded children, (ii) to establish whether these stresses occur more frequently in parents of mentally retarded children than in those of normal children, and (iii) to find any correlation between the severity of perceived stressors and the anxiety state of these parents.

## METHODS

This study was conducted in the Child Guidance Clinic at the Institute of Psychiatry and Human Behaviour, Goa, from January 2000 to February 2001. One hundred eighty subjects were selected and categorized into three groups: A, B and C. Only children who had both parents were included in this study. All the children were matched for age and sex, but not IQ.

Group A consisted of 60 parents (30 mothers and 30 fathers) of profound to moderately mentally retarded children (mean IQ: 38.63). Group B consisted of 60 parents (30 mothers and 30 fathers) of mild to borderline mentally retarded children (mean IQ: 63.2). Group C consisted of 60 parents (30 mothers and 30 fathers) of physically healthy children with normal intelligence (mean IQ: 107.7) from a city primary school.

All the mentally retarded children were diagnosed by a consultant psychiatrist using the ICD-10 criteria^8^ and their IQs were assessed by a psychologist using the following intelligence tests: the Vineland Social Maturity Scale,^9^ Seguine Form Board,^10^ Binet Kamat Test of Intelligence^11^ and the Coloured Progressive Matrices Test.^12^

A semi-structured proforma was prepared for this study, which included (i) specific variables of the child such as age, sex and severity of mental retardation (ii) sociodemographic variables such as parental age, religion, education, occupation, family income and type of family.

Each parent was interviewed and evaluated separately. Parents were then administered the Family Interview for Stress and Coping (FISC) in Mental Retardation, Section 1, of Girimaji *et al*.^13^ It is a semi-structured interview schedule, a tool to study stress and coping in families of children with mental retardation. The tool consists of section 1 for measuring perceived stress (daily care, emotional, social and financial) and section 2 for measuring mediators or coping strategies (awareness, attitudes, expectations, rearing practices and social support).

Section 1 was used in this study. It has 11 subscales which include extra inputs of care, decreased leisure time, neglect of others, disturbed behaviour, personal distress, marital problems, other interpersonal problems, effect on sibs and other family worries, altered social life, social embarrassment and financial implications. The parents were rated on a 5-point scale (no or minimal stress to a very high level of stress). The absence of stress was rated as zero and a score of four was given for a very high level of stress.

The Hamilton Anxiety Rating Scale (HARS)^14^ was used to evaluate the rate of anxiety. This scale has 14 subscales which include anxious mood, tension, fears, insomnia, intellectual (cognitive), depressed mood, somatic (muscular), somatic (sensory), cardiovascular symptoms, respiratory symptoms, gastrointestinal symptoms, genitourinary symptoms, autonomic symptoms and behaviour at interview. All the parents were rated on a 5-point scale. The absence of symptoms was rated as zero and a score of four was given for the severe category. Statistical analysis was done using the SPSS software (chi-square test, *t* test and ANOVA).

Some interesting trends were observed in the sociodemographic characteristics of the study groups. The mean age of fathers in group B was 38.27 years (SD±0.83) and it was higher as compared to that of those in groups A (36.03 years; SD±1.44) and C (34.37 years; SD±0.89). The mean age of mothers in group C (27.9 years; SD±1.07) was comparatively lower than the mean ages of those in groups A (31.3 years; SD±1.77) and B (31.3 years; SD±0.84). More than 60% of the parents in all the three groups belonged to nuclear families. The educational level of both the parents in groups A and B was lower as compared to that of group C. In all the groups, the majority of mothers were housewives. Ten per cent of fathers in group A, 3.33% in group B and 6.67% in group C were unemployed; 40% of fathers in group C, 30% in group B and 16.67% in group A were engaged in professional jobs. The family income of group C participants was higher as compared to those in groups A and B (33.33%).

## RESULTS

Table 1 compares the FISC scores of the mothers and fathers in each group separately and indicates that the scores of mothers in groups A and C differed significantly from their spouses (p<0.01 and p<0.05, respectively), whereas no significant difference was found in the FISC scores of mothers and fathers in group B.

**Table 1:** Comparison between the FISC scores of mothers and fathers in all groups

Group	FISC scores of mothers (*n*=30) Mean±SD	FISC scores of fathers (*n*=30) Mean±SD	*t*	p
A	25.46±10.92	15.10±11.22	4.99	<0.01
B	11.23±6.36	10.53±5.89	0.44	NS
C	1.50±2.06	1.07±1.82	1.94	<0.05

FISC: Family Interview for Stress and Coping; NS: not significant

Table 2 compares the perceived stressors among parents in groups A, B and C. When groups A and B were compared, a significant difference was noted in all the perceived stressors except marital problems, other interpersonal problems, effects on sibs and other family members, and financial implications. A significant difference was noted in all the perceived stressors when groups A and C were compared. When groups B and C were compared, a significant difference was noted in all the perceived stressors except other interpersonal problems.

**Table 2:** Comparison of perceived stressors among parents in groups A, B and C

Perceived stressor	A vs B	B vs C	A vs C
Extra inputs of care	p<0.001	p<0.001	p<0.001
Decreased leisure time	p<0.001	p<0.001	p<0.001
Neglect of others	p<0.001	p<0.001	p<0.001
Disturbed behaviour	p<0.001	p<0.001	p<0.001
personal distress	p<0.001	p<0.001	p<0.001
Marital problems	NS	p<0.001	p<0.001
Other interpersonal problems	NS	NS	p<0.001
Effect on sibs and other family members	NS	p<0.001	p<0.001
Altered social life	p<0.001	p<0.001	p<0.001
Social embarrassment	p<0.001	p<0.001	p<0.001
Financial implications	NS	p<0.001	p<0.001

NS: not significant

Table 3 compares the HARS scores of mothers and fathers in the groups separately and indicates that only mothers in group A differed significantly from their spouses (p<0.01), whereas no significant difference was observed in the HARS scores of mothers and fathers in groups B and C.

**Table 3:** Comparison between the HARS scores of mothers and fathers in all groups

Group	HARS scores of mothers (*n*=30) Mean±SD	HARS scores of fathers (*n*=30) Mean±SD	*t*	p
A	22.90±6.55	7.60±6.52	9.07	<0.01
B	6.03±4.28	4.67±3.46	1.36	NS
C	2.87±3.26	1.80±2.20	1.49	NS

HARS: Hamilton Anxiety Rating Scale; NS: not significant

Table 4 compares the FISC and HARS scores of groups A, B and C using ANOVA. A significant difference was noted in the perceived stress and anxiety level among parents in groups A, B and C.

**Table 4:** Comparison of the FISC and HARS scores of all the groups using ANOVA

	F	df	p
FISC score of mothers	79.76	2,87	<0.0001
FISC score of fathers	28.14	2,87	<0.0001
HARS score of mothers	145.44	2,87	<0.0001
HARS score of fathers	12.77	2,87	<0.0001

FISC: Family Interview for Stress and Coping; HARS: Hamilton Anxiety Rating Scale; F: Fischer's exact; df: degrees of freedom

Table 5 indicates a positive correlation between perceived stress and the level of anxiety (i.e. FISC and HARS scores). The correlation was found to be significant in group A (both fathers and mothers, p<0.05 and p<0.01), group B (both mothers and fathers, p<0.01 and p<0.05) and in group C only for fathers (p<0.01).

**Table 5:** Correlation between the FISC and HARS scores of parents in groups A, B and C

Group	Parent	r	p
A	Fathers	0.336	<0.05
	Mothers	0.798	<0.01
B	Fathers	0.312	<0.05
	Mothers	0.458	<0.01
C	Fathers	0.546	<0.01
	Mothers	0.22	>0.05

## DISCUSSION

Stress among parents is not an inevitable consequence of having mentally retarded children. A combination of multiple stressors appears to predict the likelihood of the parents experiencing stress and anxiety. Stressors can be defined as those life events that will bring about a change in the family system.^15^ Our study revealed that the level of parental education and family income had an impact on the perceived stress and anxiety manifested by parents of mentally retarded children in groups A and B.

A number of investigators such as Beckman,^16^ Burden,^17^ Bradshaw and Lawton^18^ found that mothers of mentally retarded children had a high level of stress. The observations of our study that (i) both parents in group A had a high level of perceived stress, (ii) mothers in groups A, B and C differed significantly on the FISC scores, and (iii) mothers in groups A and C differed significantly from their spouses on the FISC scores, are partially in accordance with those of Beckman,^16^ Burden,^17^ and Bradshaw and Lawton.^18^ Probably, mothers who were housewives without additional help felt restricted in pursuing their social and leisure activities, and experienced more stress. Further, the finding of this study that fathers in groups A, B and C differed significantly in experiencing stress is in accordance with that of Wishart *et al*.^19^

Kumar and Akhtar^20^ found that mothers of mentally retarded children had a higher level of anxiety as compared to mothers of normal children; this was also observed in our study. This study revealed that (i) mothers in group A differed significantly from those in groups B and C, (ii) mothers in group A showed a higher level of anxiety as compared to their spouses, and (iii) fathers in group A showed a comparatively higher level of anxiety than fathers in groups B and C. No literature is available to support these results.

In addition, the finding of this study that there was a positive relationship between the perceived stress and anxiety manifested in groups A, B and C (except mothers in group C needs to be confirmed by further research.

In conclusion, demographic variables had an impact on parents in groups A and B as compared to those in group C. Multifaceted factors had made these parents more vulnerable to stress than parents in the control group. The high level of stress experienced by parents in group A could be related to subjective factors such as a feeling of being restricted, social isolation and dissatisfaction, and might have paved the way for the manifestation of anxiety symptoms.

The limitation of this study was that it was a purposive sampling. Also, the FISC was designed only for families with children having severe mental retardation and was not suitable for the parents of mild to moderately mentally retarded and normal children. Personality traits of parents that might have influenced the perception of stress were also not taken into consideration.

Considering the above-mentioned lacunae of the present study, further community-based research needs to be conducted. Intervention services for parents of mentally handicapped children need to be decentralized. This will help in providing such parents with as many skills as possible to deal with their children. The support system of these parents can be enhanced by organizing self-help groups, which can serve as vehicles for communication. Parents can share their feelings and discover means to deal with their problems. Support groups can also diminish the feeling of isolation experienced by some families with mentally retarded children.
